# Analyzing the gene expression profile of anaplastic histology Wilms’ tumor with real-time polymerase chain reaction arrays

**DOI:** 10.1186/s12935-015-0197-x

**Published:** 2015-04-20

**Authors:** Jun Lu, Yan-Fang Tao, Zhi-Heng Li, Lan Cao, Shao-Yan Hu, Na-Na Wang, Xiao-Juan Du, Li-Chao Sun, Wen-Li Zhao, Pei-Fang Xiao, Fang Fang, Li-xiao Xu, Yan-Hong Li, Gang Li, He Zhao, Jian Ni, Jian Wang, Xing Feng, Jian Pan

**Affiliations:** Department of Hematology and Oncology, Children’s Hospital of Soochow University, Suzhou, China; Department of Gastroenterology, the 5th Hospital of Chinese PLA, Yin chuan, China; Department of Cell and Molecular Biology, Cancer Institute (Hospital), Chinese Academy of Medical Sciences, Peking Union Medical College, Beijing, China; Translational Research Center, Second Hospital, The Second Clinical School, Nanjing Medical University, Nanjing, China

**Keywords:** Pediatric anaplastic histology Wilms’ tumor, Real-time PCR array, Ingenuity pathway analysis, HDAC7, TP53, TGFβ1

## Abstract

**Background:**

Wilms’ tumor (WT) is one of the most common malignant neoplasms of the urinary tract in children. Anaplastic histology (unfavorable histology) accounts for about 10% of whole WTs, and it is the single most important histologic predictor of treatment response and survival in patients with WT; however, until now the molecular basis of this phenotype is not very clearly.

**Methods:**

A real-time polymerase chain reaction (PCR) array was designed and tested. Next, the gene expression profile of pediatric anaplastic histology WT and normal adjacent tissues were analyzed. These expression data were anlyzed with Multi Experiment View (MEV) cluster software further. Datasets representing genes with altered expression profiles derived from cluster analyses were imported into the Ingenuity Pathway Analysis Tool (IPA).

**Results:**

88 real-time PCR primer pairs for quantitative gene expression analysis of key genes involved in pediatric anaplastic histology WT were designed and tested. The gene expression profile of pediatric anaplastic histology WT is significantly different from adjacent normal controls; we identified 15 genes that are up-regulated and 16 genes that are down-regulated in the former. To investigate biological interactions of these differently regulated genes, datasets representing genes with altered expression profiles were imported into the IPA for further analysis, which revealed three significant networks: Cancer, Hematological Disease, and Gene Expression, which included 27 focus molecules and a significance score of 43. The IPA analysis also grouped the differentially expressed genes into biological mechanisms related to Cell Death and Survival 1.15E^−12^, Cellular Development 2.84E^−11^, Cellular Growth and Proliferation 2.84E^-11^, Gene Expression 4.43E^−10^, and DNA Replication, Recombination, and Repair 1.39E^−07^. The important upstream regulators of pediatric anaplastic histology WT were TP53 and TGFβ1 signaling (*P* = 1.15E^−14^ and 3.79E^−13^, respectively).

**Conclusions:**

Our study demonstrates that the gene expression profile of pediatric anaplastic histology WT is significantly different from adjacent normal tissues with real-time PCR array. We identified some genes that are dysregulated in pediatric anaplastic histology WT for the first time, such as HDAC7, and IPA analysis showed the most important pathways for pediatric anaplastic histology WT are TP53 and TGFβ1 signaling. This work may provide new clues into the molecular mechanisms behind pediatric anaplastic histology WT.

**Electronic supplementary material:**

The online version of this article (doi:10.1186/s12935-015-0197-x) contains supplementary material, which is available to authorized users.

## Background

The genetics of Wilms’ tumor (WT), a pediatric malignancy of the kidney, is complex. This disease is named after Dr. Max Wilms, a German surgeon (1867–1918) who firstly described this disease [1,2]. WT is a malignant tumor containing metanephric blastema, epithelial derivatives and stromal. Characteristic of this tumor is the presence of glomeruli and abortive tubules surrounded by a spindle cell stroma . The stroma consists of striated muscle, cartilage, bone, adipose tissue, fibrous tissue composition [1-3]. Clinically, the tumor compresses the kidney parenchyma, inhibiting normal function. In most children, the causes of WT are unknown. Very rarely, children that develop WT have other specific conditions present at birth, mainly congenital malformations. Moreover, mutations in *WT1* on chromosome 11p13 are observed in about 20% of WTs [4]. At least 50% of the wilm tumor patients with *WT1* mutations also carry mutations in *CTNNB1*, which encoding a proto-oncogene beta-catenin [5-7]. Although until now this disease has been curable with good long-term survival, the combination of chemotherapy, surgery and radiotherapy always results in severe complications. Novel therapeutic strategies which can decrease treatment burden and improve outcome of high risk patients are still required.

About 10% of WTs presentd as anaplastic histology (unfavorable histology). Clinical study showed that anaplastic histology is an important histologic predictor of treatment response and survival in patients with WT; the overall survival rate for patients with favorable histology is higher than for those with anaplastic unfavorable histology [8]. For anaplasia there are two histologic criteria, both of which must be present for the diagnosis: the presence of multipolar polyploid mitotic figures with marked nuclear enlargement and hyperchromasia [9]. Mutations in the p53 gene consistent with changes on chromosome 17p have been associated with foci of anaplastic histology [10-12].

Histopathology reports showed that p53 mutations were present in eight of eleven anaplastic WTs, p53 alterations may provide a molecular marker for anaplastic WTs [12].

All of this phenomenon support the hypothesis that anaplasia tumor cells evolves from a subpopulation of WT cells that have acquired additional genetic lesions as a late event.

Studies have shown that anaplasia correlates best with responsiveness to therapy. It is most consistently associated with poor prognosis when it is diffusely distributed and when identified at advanced stages. An objective of the fifth National Wilms’ Tumor Study (NWTS-5) was to evaluate the efficacy of treatment regimens for anaplastic histology Wilms’ tumor (AH). A total of 2,596 patients with Wilms’ tumor were enrolled onto NWTS-5 and the prognosis for patients with stage I AH is worse than that for patients with stage I FH(favorable histology). Four-year event-free survival (EFS) estimates for assessable patients with stage I AH (n = 29) were 69.5% (95% CI, 46.9 to 84.0). In comparison, 4-year EFS estimates for patients with stage I favorable histology (FH; n = 473) were 92.4% (95% CI, 89.5 to 94.5) [9,13]. Another report from china also showed that four-year overall survival rate for cases of favorable histology (85.8%) was higher than for those with anaplastic histology (71.4%; p = .028) [8]. Faria P and colleagues showed that the progress of Focal anaplasia (FA) is much better than diffuse anaplasia (DA) [14]. In 165 cases with anaplastic WT, Only three relapses and one death occurred among 39 cases with FA and 22 of 23 children with stage IV DA WT died of tumor. Anaplastic histology Wilms’ tumors are more resistant to chemotherapy drugs which traditionally used in children with favorable histology WT. Despite this association with therapy resistance and poor prognosis, the molecular basis of this phenotype is still unclear.

Gene expression profiles of pediatric anaplastic histology WT are vague. However, several differentially expressed genes, e.g., *MYCN, CTGF, TRIM22, CENPF, RARRES3*, and *EZH2*, have been reported to be associated with WT progression [15]. CCAAT/enhancer binding protein beta (C/EBPB), is highly expressed in both primary relapsing tumors and metastatic tumors and it is a critical survival factor for WT cells [16]. Mutations in *CTNNB1* (beta-catenin) have also been detected in a subset of pediatric WTs. The comparison between WTs with and without *CTNNB1* mutations revealed several target genes specifically deregulated in *CTNNB1*-mutated WTs [6].

Our previous work showed that a real-time polymerase chain reaction (PCR) array system is the ideal tool for analyzing the expression of a focused panel of genes [17,18]. The simplicity, flexibility, and convenience of standard SYBR green PCR detection methodology make the PCR array system accessible for routine use in any research laboratory [19]. Ingenuity Pathway Analysis (IPA) is one of several tools available to systems biology researchers and bioinformaticians in drug discovery and institutional research. IPA tool allows identification of biological networks, global functions, and functional pathways of a particular dataset. As previously mentioned, the gene expression profile of pediatric anaplastic histology WTs is still unclear. In this study, we analyzed dysregulated genes and pathways in pediatric anaplastic histology WTs with the powerful real-time PCR array platform.

## Results and discussion

### Designing the real-time PCR array

**Figure 1:**
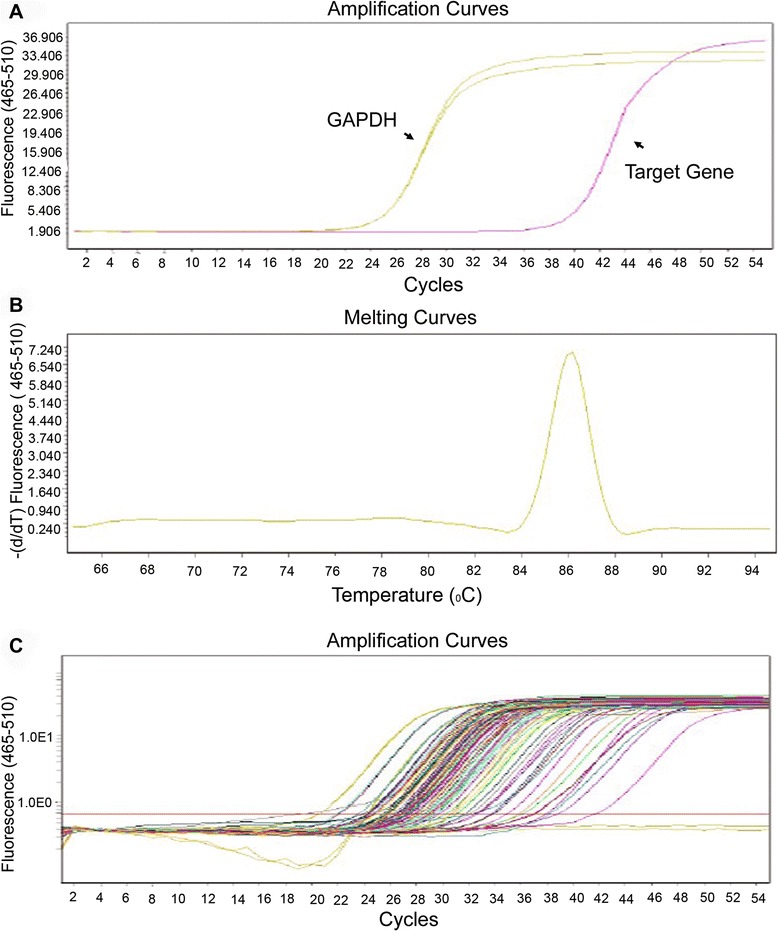
Real-time PCR array design. **(A)** Amplification of target genes and glyceraldehyde 3-phosphate dehydrogenase in real-time PCR arrays. Reactions were run on a Lightcycler 480 (Roche) using = universal thermal cycling parameters: 95°C 5 minutes, 45 cycles of 10 seconds at 95°C, 20 seconds at 60°C, and 15 seconds at 72°C. **(B)** Melting curve analysis of the PCR product of a target gene. Melting curves were obtained using the cycling parameters: 10 seconds at 95°C, 60 seconds at 60°C, and continuous melting. **(C)** Amplification of all genes in the PCR array.

88 real-time PCR primer pairs for a quantitative gene expression analysis of key genes involved in pediatric Wilms’ tumor were designed and tested (Additional file 1) [20-22]. Briefly, we assayed the expression of 11 genes from the HOX family, 15 apoptosis related genes, eight histone deacetylases, seven chemokines, 13 tumor related genes and 17 important genes in cancer. Each gene was tested via expression and melting curve analysis to ensure that the primer was specific for the target gene (Figure 1).

### Testing the real-time PCR array

Using real-time PCR, we can easily and reliably analyze the expression of a focused panel of genes involved in pediatric Wilms’ tumor. The average coefficient of variation (CV) for the CT values generated from all PCR arrays was 0.73% with replicate measurements for CT values below 30 within 0.73 cycle average standard deviation, demonstrating good inter-run reproducibility.

### Expression profile analysis of pediatric anaplastic histology WT and adjacent normal tissues

**Figure 2:**
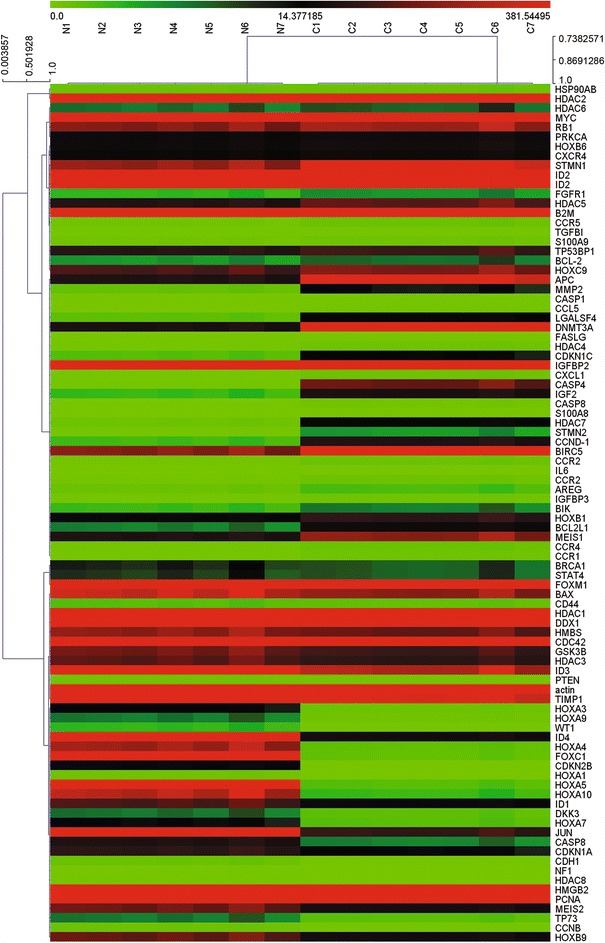
Expression and cluster analysis of pediatric anaplastic histology Wilms tumor and normal tissues adjacent to cancer. Cluster analysis of the data was performed with gene clustering from the real-time PCR arrays. For gene expression quantification, we used the comparative Ct method. First, gene expression levels for each sample were normalized to the expression level of the housekeeping gene encoding glyceraldehyde 3-phosphate dehydrogenase within a given sample (−ΔCt). The relative expression of each gene was calculated using the equation: 10^6^ *Log_2_ (−ΔCt). Gene expression between pediatric anaplastic histology Wilms tumor and normal tissues adjacent to cancer were analyzed using Multi Experiment View (MEV) cluster software.

**Figure 3:**
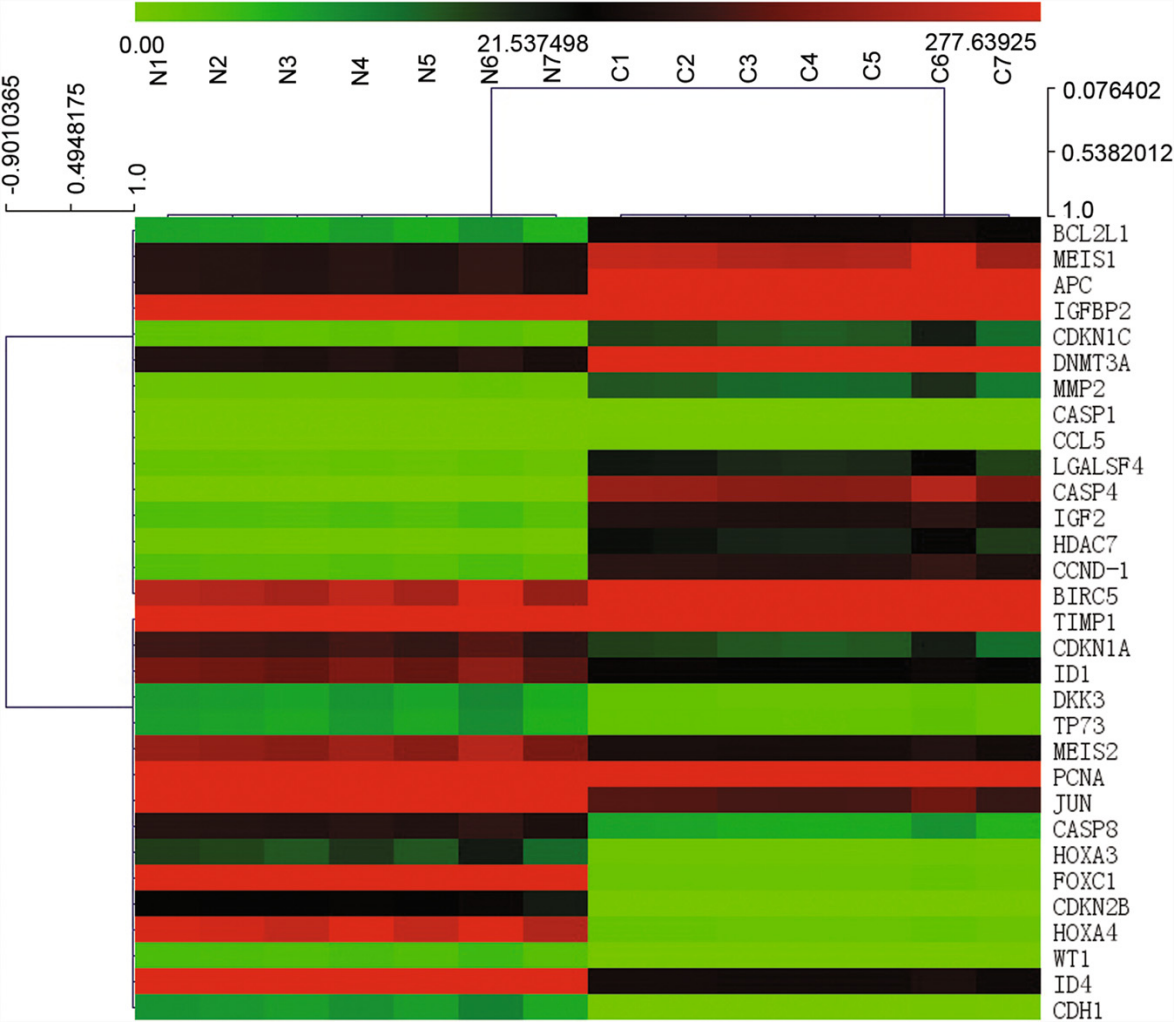
Cluster analysis of pediatric anaplastic histology Wilms’ tumor and normal tissues adjacent to cancerous tissue. Thirty one genes were successfully clustered in pediatric anaplastic histology Wilms’ tumor and normal tissues adjacent to cancerous tissue.

**Figure 4:**
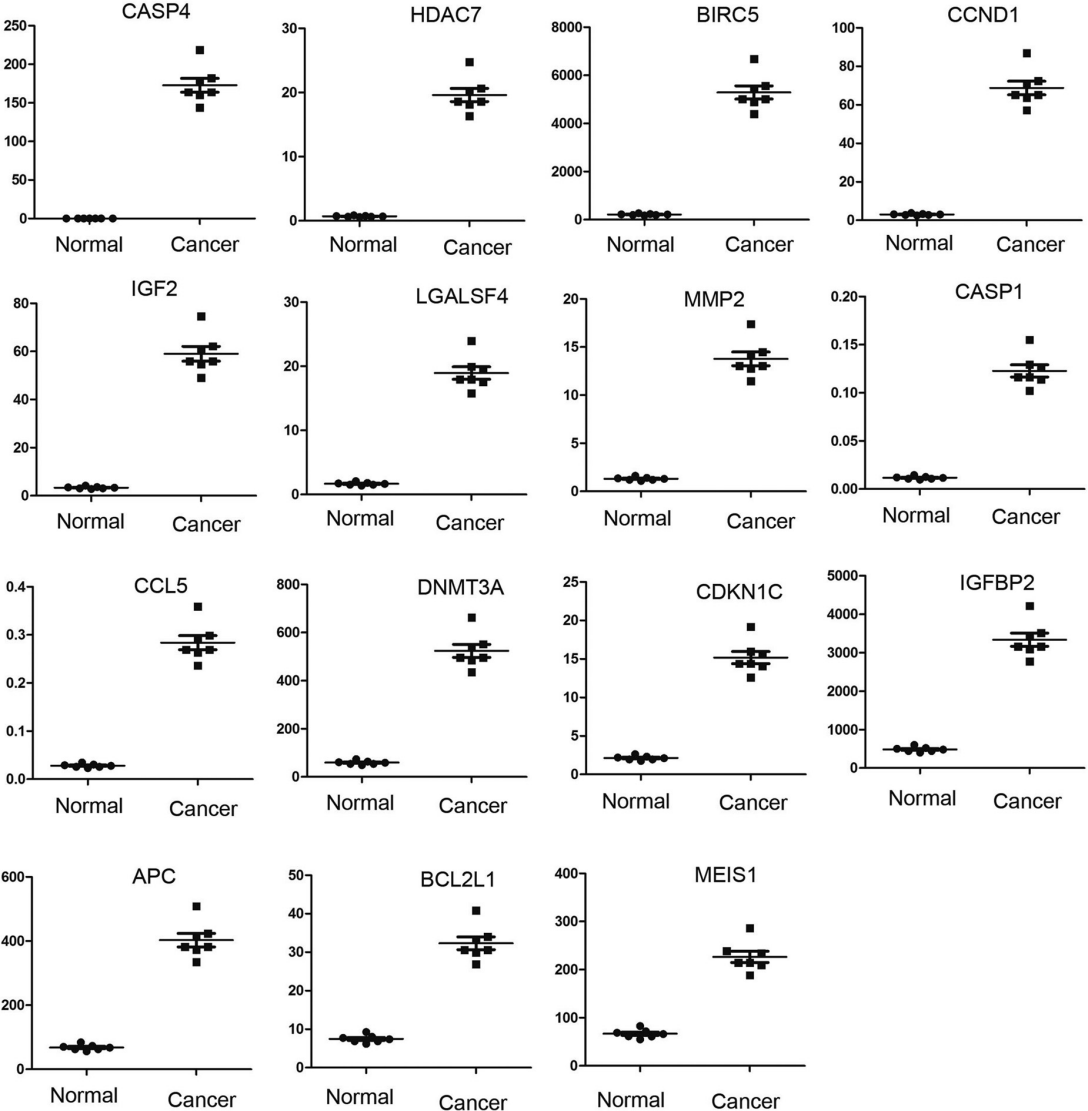
Expression of up-regulated genes in pediatric anaplastic histology Wilms’ tumor. The expression of genes in pediatric anaplastic histology Wilms’ tumor samples compared with normal tissues adjacent to cancerous tissue is presented as means ± standard error. A *P* < 0.05 was considered statistically significant.

**Figure 5:**
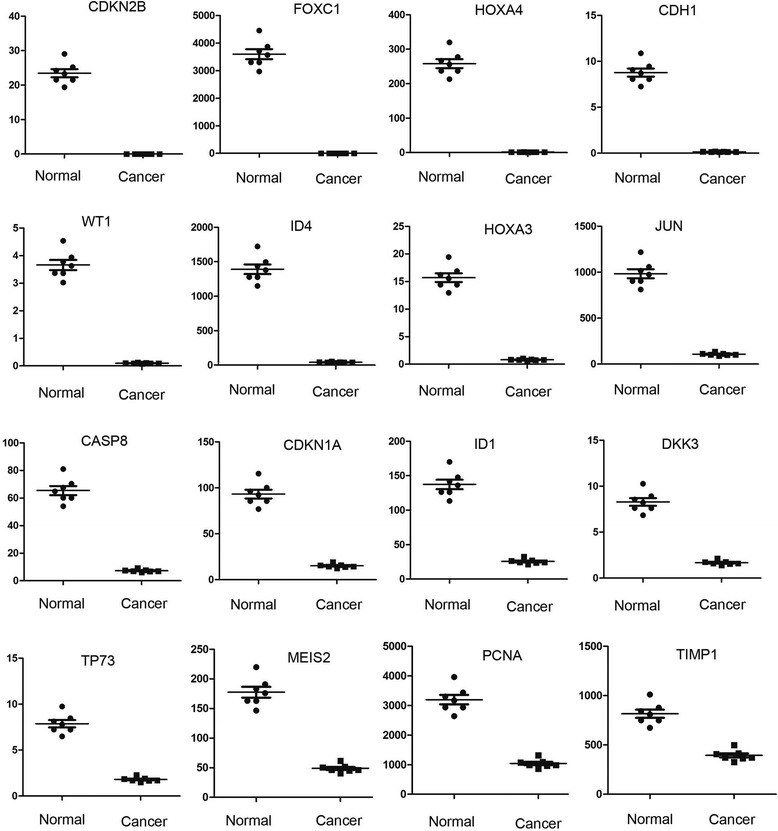
Expression of down-regulated genes in pediatric anaplastic histology Wilms tumor. The expression of genes in pediatric anaplastic histology Wilms’ tumor samples compared with normal tissues adjacent to cancerous tissue is presented as means ± standard error. A *P* < 0.05 was considered statistically significant.

Gene expression profile of pediatric anaplastic histology WT and adjacent normal control samples was analyzed with our real-time PCR arrays (Figure 2A). Clinical information from seven anaplastic pediatric WT samples used is listed in Table 1. After obtaining original data, we analyzed expression data with Multi Experiment View (MEV) cluster software. The gene expression profile of pediatric anaplastic histology WT was significantly different from normal tissues adjacent to cancer, and comprised a set of genes that could be successfully clustered (Figure 3). Compared with normal controls, we identified 15 genes that were up-regulated and 16 genes that were down-regulated in pediatric anaplastic histology WT. The detailed expression of each up-regulated gene in pediatric anaplastic histology WT is presented in Figure 4 and Table 2, while the expression of down-regulated genes is presented in Figure 5 and Table 3. Figure 2 shows the 88 genes detected by PCR array can distinguish anaplastic Wilms tumor and adjacent normal tissues, Figure 3 shows that use a small group of different genes (fold change > 2.0) can also distinguish anaplastic Wilms tumor and adjacent normal tissues.

**Table 1:** **Pediatric anaplastic histology Wilms’ tumor patients’ clinical features**

	**Age (year)**	**Sex**	**Hb (g/L)**	**WBC (10** ^**9**^ **/L)**	**RBC (10** ^**12**^ **/L)**	**PLt (10** ^**9**^ **/L)**	**Diagnosis**
1	1	M	22.5	8.9	4.47	252	Wilm’s Tumor
2	3	M	100	16.9	3.8	326	Wilm’s Tumor
3	2.5	F	115	7.4	4.47	231	Wilm’s Tumor
4	1.7	F	94	16.7	3.32	599	Wilm’s Tumor
5	0.8	M	127	8.3	4.8	312	Wilm’s Tumor
6	2.7	F	106	7.7	3.86	161	Wilm’s Tumor
7	2.9	F	94	3.4	2.98	201	Wilm’s Tumor

Hb: Hemoglobin; WBC: White blood cells; RBC: Red blood cells; PLt: Platelet.

**Table 2:** **Genes up-regulated in the pediatric Wilms’ tumor compared with normal tissues adjacent to cancerous tissue**

	**Gene**	**Description**	**Control**	**WT**	**Fold change**	**P**
1	CASP4	Caspase 4	0.145779	172.8901	1185.974	<0.001
2	HDAC7	Histone deacetylase 7	0.703126	19.61264	27.89349	0.0011
3	BIRC5	Baculoviral IAP repeat containing 5	217.2588	5288.839	24.3435	0.0021
4	CCND1	Cyclin D1	3.035334	68.77021	22.65655	0.0023
5	IGF2	Insulin-like growth factor 2	3.39134	59.04372	17.41014	0.0025
6	LGALS4	Lectin, galactoside-binding, soluble, 4	1.683957	18.94456	11.25002	0.0028
7	MMP2	Matrix metallopeptidase 2	1.31208	13.77244	10.49664	0.0028
8	CASP1	Caspase 1	0.011731	0.122743	10.46301	0.0031
9	CCL5	Chemokine (C-C motif) ligand 5	0.0282	0.283951	10.06905	0.0031
10	DNMT3A	DNA methyltransferase 3 alpha	59.37795	524.1049	8.826591	0.0033
11	CDKN1C	Cyclin-dependent kinase inhibitor 1C	2.13148	15.17592	7.119897	0.0033
12	IGFBP2	Insulin-like growth factor binding protein 2	485.0049	3335.557	6.877367	0.0034
13	APC	Adenomatous polyposis coli	68.20736	402.7419	5.904669	0.0054
14	BCL2L1	BCL2-like 1	7.47387	32.30559	4.322471	0.0059
15	MEIS1	Meis homeobox 1	66.80367	226.5541	3.391342	0.0059

**Table 3:** **Genes down-regulated in the pediatric Wilms tumor compared with normal tissues adjacent to cancerous tissue**

	**Gene**	**Description**	**Control**	**WT**	**Fold change**	**P**
1	CDKN2B	Cyclin-dependent kinase inhibitor 2B (p15)	23.45552	0.002128	−11022.3	<0.001
2	FOXC1	Forkhead box C1	3595.198	1.408063	−2553.29	0.0011
3	HOXA4	Homeobox A4	258.1124	1.562344	−165.208	0.0011
4	CDH1	Cadherin 1, type 1, E-cadherin	8.779151	0.157531	−55.7295	0.0012
5	WT1	Wilms’ tumor 1	3.66003	0.099698	−36.7111	0.0013
6	ID4	Inhibitor of DNA binding 4	1390.951	41.75022	−33.316	0.0014
7	HOXA3	Homeobox A3	15.69089	0.814345	−19.2681	0.0017
8	JUN	Jun proto-oncogene	983.5506	107.912	−9.11437	0.0017
9	CASP8	Caspase 8	65.42887	7.329485	−8.9268	0.0021
10	CDKN1A	cyclin-dependent kinase inhibitor 1A (p21)	93.174	15.17592	−6.1396	0.0021
11	ID1	Inhibitor of DNA binding 1	137.3636	25.70027	−5.34483	0.0034
12	DKK3	Dickkopf 3 homolog	8.292778	1.686125	−4.91825	0.0043
13	TP73	Tumor protein p73	7.868963	1.807144	−4.35436	0.0056
14	MEIS2	Meis homeobox 2	177.5222	48.96611	−3.62541	0.0061
15	PCNA	Proliferating cell nuclear antigen	3195.565	1040.969	−3.0698	0.0067
16	TIMP1	TIMP metallopeptidase inhibitor 1	815.6778	394.4536	−2.06787	0.0078

Results showed that some of the dysregulated genes that we identified are consistent with previous other’s reports, such as *BIRC5, IGF2*, and *IGFBP2. BIRC5*, also known as survivin, is expressed in virtually most of human tumors, but is undetectable or present at very low levels in most normal adult tissues. This tumor-specific expression of survivin is predominantly dictated at the level of transcription, as survivin gene expression may be globally deregulated in tumors *in vivo*. Immunohistochemical expression of survivin was analyzed in 59 cases of primary WT and in ten normal kidney specimens, taken from the same patients, but distant from the tumor. Decreased cytoplasmic or nuclear overexpression of survivin may be related to a favorable WT prognosis [23]. Furthermore, treatment with YM155, a novel survivin inhibitor, resulted in inhibition of SK-NEP-1 proliferation, illustrating its potential as a therapeutic modality in WT [24].

Human insulin-like growth factor II (IGF2) is overexpressed and its imprinting is disrupted in many tumors, including WT. In WTs that demonstrate maintenance of imprinting of IGF2, IGF2-AS (the anti-sense version of IGF2) is also imprinted [25]. A pilot study indicated a significant relationship between loss of imprinting of IGF2 and family history as well as personal history of colorectal cancer, suggested that loss of IGF2 imprinting might be a valuable molecular marker of predicting an individual’s risk for colon cancer [26]. Loss of imprinting of IGF2 results in a modest increase in IGF2 expression, and it is present in about 30% of patients with colorectal cancer. To investigate its role in intestinal tumorigenesis, a mouse model of IGF2 loss of imprinting was created by crossing female H19^+/−^ mice with male Apc^+^/Min mice. Mice with loss of imprinting developed twice as many intestinal tumors as did control littermates. Thus, altered maturation of non-neoplastic tissue may be one mechanism by which epigenetic changes increase cancer risk [27]. Mice with sustained mosaic, somatic ablation of *WT1* and constitutional IGF2 up-regulation have been engineered, mimicking a subset of human tumors; mice with this combination of genetic alterations developed tumors at an early age. Mechanistically, increased IGF2 expression up-regulates ERK1/2 phosphorylation [28].

Insulin-like growth factors binding protein-2 (IGFBP-2) has been measured in plasma of children with WT and was shown to be significantly elevated relative to controls. Therefore, IGFBP-2 measurements might be valuable as a marker for monitoring this type of tumor, either as an adjunct to diagnosis or for tumor growth surveillance during therapy [29]. IGFBP2 overexpression has also been noted in other cancers, such as in ovarian malignant tissues and in the serum and cystic fluid of ovarian cancer patients. Moreover, IGFBP2 was significantly overexpressed in invasive serous ovarian carcinomas compared with borderline serous ovarian tumors and IGFBP2 has been shown to enhance the invasion capacity of ovarian cancer cells. Furthermore, plasma IGFBP-2 is elevated in patients with malignant adrenocortical tumors and the major factor affecting IGFBP-2 levels in these patients is tumor stage [30]. Blockade of IGFBP2 may thus constitute a viable strategy for targeted cancer therapy, including WT [31].

Our work also identified novel dysregulated genes in pediatric anaplastic histology WT. For instance, gene expression profiling showed that *HDAC7* is dysregulated in pediatric anaplastic histology WT [18]. *HDAC7* is a crucial player in cancer cell proliferation, as knockdown of *HDAC7* results in significant G_1_/S arrest in different cancer cell lines. *HDAC7* silencing blocked cell cycle progression by suppressing c-Myc expression and increasing p21 and p27 protein levels [32]. Furthermore, acute lymphoblastic leukemia (ALL) samples showed higher expression levels of HDAC7 compared to normal bone marrow samples. *HDAC7* expression levels higher than median values were also associated with a lower 5-year event-free survival (EFS) in the overall group. Moreover, higher expression of *HDAC7* is associated with poor prognosis in childhood ALL and could be a promising therapeutic target for the treatment of refractory childhood ALL [33]. *HDAC7* has also been implicated in pancreatic tumors; for instance, increased *HDAC7* expression discriminates pancreatic tumors from normal pancreas tissue [34]. Silencing of *HDAC7* in endothelial cells alters their morphology, migration, and capacity to form capillary tube-like structures *in vitro* but does not affect cell adhesion, proliferation, or apoptosis. In addition, *HDAC7* is a key modulator of endothelial cell migration and hence angiogenesis [35]. In other cancers, *HDAC7* is up-regulated, such as in early human colon carcinogenesis in which it plays an important role in early carcinogenic events [36]. In chronic lymphocytic leukemia patients, *HDAC7* was a treatment-free survival independent predictor and poor prognosis was associated with *HDAC7* overexpression [37]. In the present study, we demonstrate for the first time that *HDAC7* is up-regulated in anaplasia pediatric WT, suggesting that it may play an important role in the occurrence and development of this disease. As with the other cancers mentioned previously, *HDAC7* is a potential candidate therapeutic target for anaplasia WTs.

### Ingenuity pathway analysis of the dysregulated genes in pediatric anaplastic histology WT

**Figure 6:**
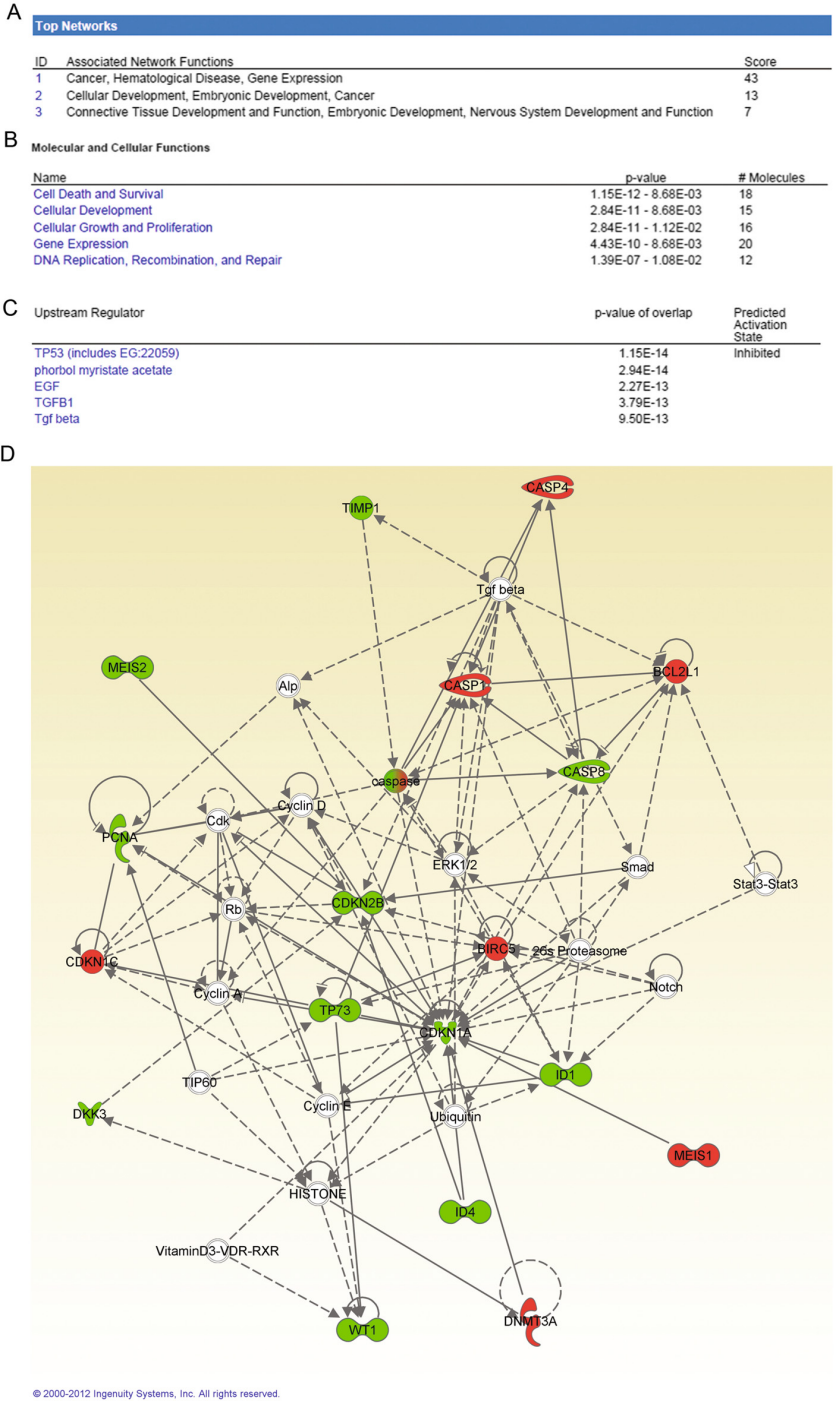
Ingenuity Pathways Analysis summary. **(A)** A list of the top two networks with their respective scores obtained from the Ingenuity Pathway Analysis (IPA). **(B)** A list of molecular and cellular functions with their respective scores obtained from the IPA. **(C)** Upstream regulator list that includes TP53 and TGFβ1. IPA analysis showed in pediatric anaplastic histology Wilms’ tumor the important upstream regulators are TP53 and TGFβ1 signaling. **(D)** Most highly rated network in the IPA analysis. The genes that are shaded were determined to be significant based on statistical analysis. The solid line represents a direct interaction between the two gene products and the dotted line indicates an indirect interaction.

To investigate possible biological interactions of differently regulated genes, datasets representing genes with altered expression profiles derived from the real-time PCR array analyses were imported into the IPA tool. The list of differentially expressed genes analyzed by IPA revealed 12 significant networks (Additional file 2) and Figure 6A shows the top three networks identified by IPA: Cancer, Hematological Disease, and Gene Expression comprised 27 focus molecules and significance scores of 43 (Figure 6D). The score represents the probability that a collection of genes equal to or greater than the number in a network could be achieved by chance alone. A score of three indicates a 1/1,000 chance that the focus genes are in a network not due to random chance. The IPA analysis also groups differentially expressed genes into biological mechanisms that are related to Cell Death and Survival 1.15E^−12^, Cellular Development 2.84E^−11^, Cellular Growth and Proliferation 2.84E^−11^, Gene Expression 4.43E^−10^, and DNA Replication, Recombination, and Repair 1.39E^−07^ (Figure 6B). IPA analysis revealed that, in pediatric anaplastic histology WT, the important upstream regulators are p53 and *TGFβ1* (Figure 6C).

Tumor protein p53, also known as p53, is a protein that in humans is encoded by the *TP53* gene. The p53 protein plays a crucial role in multicellular organisms, in which it regulates the cell cycle and, thus, functions as a tumor suppressor. Mutations in p53 frequently occur in WT [38-42]. Although a correlation between anaplasia and mutations in the p53 tumor suppressor gene have been previous identified in WT, the prognostic significance of p53 in WT remains largely unresolved. Statistical analyses have revealed significant correlations between p53 expression and anaplasia and survival. Therefore, the *TP53* gene may be of prognostic relevance with respect to poor outcome when present in WT unfavorable histology [43]. In another study, *TP53* mutations were present in 8 of 11 anaplastic WTs. Thus, *TP53* alterations act as a molecular marker for anaplastic WTs [12].

TGFβ1 is a polypeptide member of the transforming growth factor beta superfamily of cytokines. It is a secreted protein that performs many cellular functions, including controlling cell growth, proliferation, differentiation, and apoptosis [44]. Expression of TGFβ1 in WT is associated with disease progression [45]: immunohistochemistry was used to examine TGFβ1 expression in 51 primary tumors and 17 invasive/metastatic tumors and the positive expression rate was 50.98% (26/51) and 82.35% (14/17), respectively. Furthermore, positive expression of TGFβ1 in WT correlated with tumor invasion and disease progression, which might be useful for identifying patients at high risk of unfavorable outcomes [45]. To date, the possible regulation of TP53 and TGFβ1 signaling in pediatric anaplastic histology WT has not been reported. Future studies should focus on the molecular mechanism of TP53 and TGFβ1 in WT.

## Conclusions

The present study demonstrates that the gene expression profile of pediatric anaplastic histology WT is significantly different from normal controls; there are 15 genes that are up-regulated and 16 genes that are down-regulated in pediatric anaplastic histology WT. We identified several novel genes that are dysregulated in pediatric anaplastic histology WT, such as *HDAC7*. IPA analysis showed the two most important pathways involved in pediatric anaplastic histology WT are *TP53* and *TGFβ1* signaling. This work may provide new clues into the molecular mechanisms behind pediatric anaplastic histology WT.

## Methods

### Patients and samples

Biopsy specimens were obtained from 7 patients with pediatric anaplastic histology wilm’s tumor, who presented at the Department of Hematology and Oncology, Children’s Hospital of Soochow University between 2010 and 2012. Ethical approval was provided by the Children’s Hospital of Soochow University Ethics Committee (No.SUEC2010-031), and informed consent was obtained from the parents or guardians.

Pathology slides and institutional pathology reports were reviewed by the study pathologists. The designation of anaplasia was applied to tumors with cells having major diameters at least three times those of adjacent cells, increased chromatin content (hyperchromaticity) and the presence of atypical polyploid mitotic figures.

### Real-time PCR array analysis expression profile of pediatric anaplastic histology WT and adjacent normal tissues

Most of the primers were from a database of Real-time primers, Center for Medical Genetics (http://medgen.ugent.be/CMGG/). The rest of primers were designed using the online program Primer 3 (www.fokker.wi.mit.edu/primer3/input.htm). Primer selection parameters were set to primer size: 20–26 nts; primer melting temperature: 60 to 64°C; GC clamp: 1; and product size range: generally 120–240 bp but down to 100 bp if no appropriate primers could be identified. Primers were ordered from Invitrogen. (Genes and sequence of the primers was presented in Additional file 1). Samples from each group were submerged in 2 ml Trizol (Invitrogen Co., NY, USA) for RNA extraction, stored at −80°C until further processed. cDNA synthesis was performed on 4 ug of RNA in a 10 ul sample volume using SuperScript II reverse transcriptase (Invitrogen Co., NY, USA) as recommended by the manufacturer. Real-time PCR array analysis was according to the MIQE Guidelines and performed in a total volume of 20 μl including 1 μl of cDNA, primers (0.2 mM each) and 10 μl of SYBR Green mix (Roche). Reactions were run on an Lightcycler 480 (Roche) using universal thermal cycling parameters (95°C for 5 min, 45 cycles of 10 sec at 95°C, 20 sec at 60°C and 15 sec at 72°C; followed by a melting curve: 10 sec at 95°C, 60 sec at 60°C and continued melting). The results were obtained using the sequence detection software of the Lightcycler 480 and analyzed using Microsoft Excel. For quality control purposes, melting curves were acquired for all samples. The comparative Ct method was used to quantify gene expression. The target gene expression level was normalized to expression of the housekeeping gene glyceraldehyde 3-phosphate dehydrogenase (GAPDH) within the same sample (−⊿Ct), the relative expression of each gene was calculated with 10^6^ *Log2(−⊿Ct). Statistical significance of the gene expression difference between the AML and the control samples was calculated with the *T*-test using SPSS 11.5 software and then the relative expression of each gene was calculated using Log2 (−⊿Ct).

### Ingenuity pathway analysis (IPA)

Our datasets representing genes with altered expression profile derived from array analyses, 31 different genes (fold change > 2.0) were imported into the Ingenuity Pathway Analysis Tool (IPA Tool; Ingenuity H Systems, Redwood City, CA, USA; http://www.ingenuity.com).

IPA analysis was introduced before [24]. IPA Tool allows the identification of biological networks, global functions and functional pathways of a particular dataset.

### Statistical analysis

SPSS v11.5 (SPSS Inc., Chicago, IL) was used for statistical analysis. For gene expression quantification, we used the comparative Ct method. In our PCR array analysis three replicates of each gene were analyzed. First, gene expression levels for each sample were normalized to the expression level of the housekeeping gene encoding Glyceraldehydes 3-phosphate dehydrogenase (GAPDH) within a given sample (−⊿Ct); the relative expression of each gene was calculated with 10^6^ *Log_2_(−⊿Ct). The expression of the pediatric anaplastic histology wilm’s tumor samples compared to the control samples was presented average ± SE. A *p* <0.05 was considered statistically significant.

## Additional files

Additional file 1:
**Genes and PCR primers of Real-time PCR array.**
Additional file 2:
**Summary of IPA analysis.**

## Abbreviations

WT: Wilms’ Tumor
PCR: Polymerase chain reaction
IPA: Ingenuity pathway analysis
MEV: Multi experiment view
CV: Coefficient of variation
IGF2: Human insulin-like growth factor II
ALL: Acute lymphoblastic leukemia
