# Supplementary material for: Analyzing the gene expression profile of anaplastic histology Wilms’ tumor with real-time polymerase chain reaction arrays
Source: Cancer Cell Int. 2015 Apr 20;15:44. doi: 10.1186/s12935-015-0197-x (PMC4486424; doi:10.1186/s12935-015-0197-x)
Supplement: Additional file 2: — Summary of IPA analysis. [file 12935_2015_197_MOESM2_ESM.pdf]

IPA® Summary of Analysis

Analysis Name: API-1-WT - 2012-09-07 10:33
Analysis Creation Date: 2012-09-07
Build version: 172788
Content version: 14197757 (Release Date: 2012-08-11)

Analysis settings

View

Reference set: Ingenuity Knowledge Base (Genes Only)

Relationship to include: Direct and Indirect

Includes Endogenous Chemicals

Optional Analyses: My Pathways My List

Filter Summary:

Consider only molecules and/or relationships where

(species = Uncategorized (e.g. chemicals) OR Human) AND

(confidence = Experimentally Observed) AND

(tissues/cell lines = Macrophages OR Adipose OR Monocyte-derived macrophage OR Vd2 Gamma-delta T cells OR Salivary Gland OR CD56dim NK cells OR Memory B cells OR Activated Vd1 Gamma-delta T cells OR Naive B cells OR Placenta OR Plasmacytoid dendritic cells OR Liver OR Jurkat OR Cytotoxic T cells OR Mammary Gland OR SR OR Spleen OR Natural T-regulatory cells OR Activated helper T cells OR Naive helper T cells OR BDCA-1+ dendritic cells OR Skeletal Muscle OR HL-60 OR Central memory cytotoxic T cells OR Pancreas OR Testis OR Kidney OR K-562 OR Other Tissues and Primary Cells OR Prostate Gland OR RAW 264.7 OR Activated CD56bright NK cells OR MOLT-4 OR Thymus OR Epidermis OR CD56bright NK cells OR Effector memory RA+ cytotoxic T cells OR Effector memory cytotoxic T cells OR Monocytes OR Effector T cells OR Vd1 Gamma-delta T cells OR Activated CD56dim NK cells OR Central memory helper T cells OR Neutrophils OR Lung OR Uterus OR Immature monocyte-derived dendritic cells OR Murine NKT cells OR Retina OR Large Intestine OR Effector memory

(c) 2000-2012 Ingenuity Systems, Inc. All rights reserved.

helper T cells OR THP-1 OR BDCA-3+ dendritic cells OR Th1 cells OR Activated Vd2 Gamma-delta T cells OR Th2 cells OR Small Intestine OR Mature monocyte-derived dendritic cells OR Bladder OR CCRF-CEM OR Heart OR Ovary OR Stomach) AND
(data sources = BIND OR BIOGRID OR Cognia OR DIP OR Ingenuity Expert Findings OR Ingenuity ExpertAssist Findings OR INTACT OR Interactome studies OR MINT OR MIPS OR TarBase)

Top Networks

ID	Associated Network Functions	Score
1	Cancer, Hematological Disease, Gene Expression	43
2	Cellular Development, Embryonic Development, Cancer	13
3	Connective Tissue Development and Function, Embryonic Development, Nervous System Development and Function	7

(c) 2000-2012 Ingenuity Systems, Inc. All rights reserved.

INGENUITY® SYSTEMS

Top Bio Functions

Diseases and Disorders

Name	p-value	# Molecules
Cancer	9.76E-14 - 1.11E-02	27
Reproductive System Disease	9.76E-14 - 8.68E-03	21
Respiratory Disease	5.81E-12 - 1.08E-02	14
Gastrointestinal Disease	1.21E-11 - 1.08E-02	20
Hepatic System Disease	1.21E-11 - 9.28E-03	14

Molecular and Cellular Functions

Name	p-value	# Molecules
Cell Death and Survival	1.15E-12 - 8.68E-03	18
Cellular Development	2.84E-11 - 8.68E-03	15
Cellular Growth and Proliferation	2.84E-11 - 1.12E-02	16
Gene Expression	4.43E-10 - 8.68E-03	20
DNA Replication, Recombination, and Repair	1.39E-07 - 1.08E-02	12

Physiological System Development and Function

Name	p-value	# Molecules
Tumor Morphology	1.02E-06 - 1.08E-02	8
Embryonic Development	1.78E-05 - 1.08E-02	15
Renal and Urological System Development and Function	3.27E-05 - 1.08E-02	10
Organismal Survival	5.85E-05 - 1.08E-02	7
Cardiovascular System Development and Function	9.55E-05 - 7.99E-03	10

(c) 2000-2012 Ingenuity Systems, Inc. All rights reserved.

Top Canonical Pathways

Name	p-value	Ratio
p53 Signaling	8.88E-10	7/96 (0.073)
Huntington's Disease Signaling	4.5E-06	6/232 (0.026)
Molecular Mechanisms of Cancer	5.47E-06	7/372 (0.019)
Colorectal Cancer Metastasis Signaling	8.47E-06	6/250 (0.024)
Pancreatic Adenocarcinoma Signaling	7.45E-05	4/119 (0.034)

Top Molecules

Fold Change up-regulated

Molecules	Exp. Value	Exp. Chart
APC	↑1.000	
BCL2L1	↑1.000	
BIRC5	↑1.000	
CASP1	↑1.000	
CASP4	↑1.000	
CCL5	↑1.000	
CDKN1C	↑1.000	
DNMT3A	↑1.000	
HDAC7	↑1.000	
IGF2	↑1.000	

Fold Change down-regulated

Molecules	Exp. Value	Exp. Chart
WT1	↓-1.000	
TP73	↓-1.000	
TIMP1	↓-1.000	

(c) 2000-2012 Ingenuity Systems, Inc. All rights reserved.

INGENUITY® SYSTEMS

PCNA	↓-1.000
MEIS2	↓-1.000
JUN	↓-1.000
ID4	↓-1.000
ID1	↓-1.000
HOXA4	↓-1.000
HOXA3	↓-1.000

Top Upstream Regulators

Upstream Regulator	p-value of overlap	Predicted Activation State
TP53 (includes EG:22059)	1.15E-14	Inhibited
phorbol myristate acetate	2.94E-14	
EGF	2.27E-13	
TGFB1	3.79E-13	
Tgf beta	9.50E-13	

(c) 2000-2012 Ingenuity Systems, Inc. All rights reserved.

INGENUITY® SYSTEMS

Top My Lists

Name	p-value	Ratio
Direct players of IL24	4.27E-02	1/21 (0.048)
BIOMARKERS HNC and mouse HPV16	1.89E-01	1/100 (0.01)

Top My Pathways

Name	p-value	Ratio
IL24 pic	1.5E-04	3/54 (0.056)
Networks 13,3 Merged 2	1.23E-03	2/50 (0.04)
Merged networks	1.15E-02	2/269 (0.007)
FASN malignancy pathway	1.6E-02	2/142 (0.014)

Top Tox Lists

Name	p-value	Ratio
p53 Signaling	1.03E-09	7/95 (0.074)
Cardiac Necrosis/Cell Death	3.09E-06	6/193 (0.031)
Renal Necrosis/Cell Death	1.19E-05	7/374 (0.019)
Hepatic Fibrosis	4.44E-05	4/92 (0.043)
Liver Necrosis/Cell Death	1.3E-04	5/232 (0.022)

(c) 2000-2012 Ingenuity Systems, Inc. All rights reserved.

INGENUITY® SYSTEMS

Top Tox Functions

Cardiotoxicity

Name	p-value	# Molecules
Cardiac Necrosis/Cell Death	1.88E-05 - 1.73E-02	4
Cardiac Stenosis	2.61E-03 - 1.51E-02	2
Cardiac Infarction	4.35E-03 - 4.35E-03	1
Cardiac Arrythmia	2.71E-02 - 1.64E-01	2

Hepatotoxicity

Name	p-value	# Molecules
Liver Hyperplasia/Hyperproliferation	1.21E-11 - 9.16E-02	13
Hepatocellular Carcinoma	2.24E-10 - 9.16E-02	12
Liver Necrosis/Cell Death	6.16E-06 - 2.58E-02	4
Liver Hepatitis	6.33E-04 - 8.36E-02	3
Liver Proliferation	3.56E-03 - 6.54E-02	2

Nephrotoxicity

Name	p-value	# Molecules
Renal Necrosis/Cell Death	2.86E-05 - 3.01E-02	7
Renal Proliferation	3.27E-05 - 5.10E-02	5
Glomerular Injury	1.67E-03 - 2.18E-03	2
Kidney Failure	2.18E-03 - 9.82E-03	2
Renal Inflammation	2.37E-02 - 3.64E-02	2

(c) 2000-2012 Ingenuity Systems, Inc. All rights reserved.

INGENUITY® SYSTEMS
